# Blood Neurofilament Light Chain and Phospho-Tau 181 in Subjects with Mild Cognitive Impairment Due to Age-Related Hearing Loss

**DOI:** 10.3390/jcm14030672

**Published:** 2025-01-21

**Authors:** Giuseppe Alberti, Daniele Portelli, Francesca Polito, Anita Graceffa, Laura Licitri, Sabrina Loteta, Margherita Maria Torre, Irene Gasparo, Vincenzo Rizzo, M’hammed Aguennouz, Vincenzo Macaione

**Affiliations:** 1 Department of Adult and Development Age Human Pathology, University of Messina, 98122 Messina, Italy; galberti@unime.it (G.A.); daniele.portelli09@gmail.com (D.P.); lotetas@unime.it (S.L.); 2 Department of Clinical and Experimental Medicine, University of Messina, 98122 Messina, Italy; fpolito@unime.it (F.P.); agraceffa@unime.it (A.G.); llicitri@unime.it (L.L.); tmargherita.91@gmail.com (M.M.T.); iregasparo@unime.it (I.G.); vrizzo@unime.it (V.R.); aguenoz@unime.it (M.A.)

**Keywords:** hearing loss, cognitive impairment, neurodegeneration, neurofilament, phospho-tau, hearing aids

## Abstract

**Background**: Mild cognitive impairment is increasingly recognized as a precursor to more severe neurodegenerative conditions, particularly in the context of aging. Recent studies have highlighted the intersection of hearing loss and cognitive decline, suggesting that auditory deficits may exacerbate cognitive impairments in older adults, proposing the use of hearing aids to mitigate cognitive decline, and indicating that early intervention in hearing loss could be crucial for preserving cognitive function. The underlying mechanisms of the relationship between hearing and cognitive impairment may involve neuroinflammatory processes and neurodegeneration. Recent studies have evidenced the role of tau proteins and neurofilaments as biomarkers in the onset and progression of neurodegenerative diseases. **Methods**: We selected 30 subjects with age-related hearing loss, and we evaluated their cognitive status through the administration of screening tests, which also measured neurofilament light chain and phospho-tau 181 serum levels as biomarkers of neurodegeneration. The subjects were re-evaluated six months after the hearing aid fitting. **Results**: Patients with hearing impairment presented slightly altered results on cognitive tests, typical of a mild cognitive impairment. At the same time, serum levels of neurofilament light chain and phospho-tau 181 were significantly increased compared to the matched control group. After the hearing aids fitting, auditory, cognitive, and serum values results improved. **Conclusions**: The results of the study highlight the cognitive involvement in patients with hearing impairment and identify neurofilament light chain and phospho-tau 181 as serum biomarkers of neurodegeneration useful in monitoring the pathology.

## 1. Introduction

Globally, approximately 1.5 billion people are affected by hearing loss, with 430 million of them experiencing moderate to severe hearing impairment [1]. The Global Burden of Diseases, Injuries, and Risk Factors Study, in its latest report published in 2024, estimates that hearing loss has become the fourth leading cause of years lived with disability and a major global health concern, especially among the elderly [2]. Age-related hearing loss (ARHL), also known as presbycusis, is a condition that affects elderly individuals. It is estimated to be the most common chronic sensory deficit in older adults, causing significant communication difficulties in 50% of those over the age of 70. ARHL is characterized as a progressive, bilateral, symmetric sensorineural hearing loss resulting from the effects of aging on the auditory system [3]. The prevalence of ARHL is approximately 3% in adults aged 20–29, 49% in those aged 60–69, and over 80% in individuals older than 85 years [4]. The growing attention to ARHL stems not only from it being one of the most common health conditions affecting the elderly but also from the risk of developing dementia and its relationship with mental health [5]. The use of hearing aids has emerged as a primary intervention for managing ARHL. Research indicates that hearing aids can significantly improve auditory function and quality of life for older adults. For instance, a study by Mustafa et al. demonstrated that the percentage of patients experiencing severe hearing handicaps decreased dramatically from 45.6% to 8.8% following hearing aid fitting [6]. This substantial improvement underscores the effectiveness of hearing aids in mitigating the challenges posed by ARHL. Furthermore, the benefits of amplification extend beyond mere auditory enhancement; they also encompass cognitive and emotional dimensions, as evidenced by findings that suggest hearing aids can positively influence working memory and cognitive function in older adults [7,8]. Numerous studies have shown that ARHL is associated with social isolation, frailty, and an increased risk of falls. Hearing loss can increase the likelihood of social isolation, loneliness, and depression among the elderly [4]. The Lancet International Commission on Dementia, Prevention, Intervention, and Care (2024) estimated that treating midlife hearing loss (e.g., with hearing aids) could reduce the risk of dementia, identifying hearing loss as the most significant modifiable risk factor for dementia among twelve health and lifestyle factors [9]. In recent years, efforts have been made to identify early markers of neurodegeneration. Neurofilament light chain (NfL) levels in cerebrospinal fluid (CSF) and serum correlate with neuroaxonal damage, making them valuable for diagnosing and monitoring various neurodegenerative diseases, including Alzheimer’s disease (AD), Parkinson’s disease (PD), and amyotrophic lateral sclerosis (ALS) [10]. Higher plasma NfL levels were significantly associated with MRI evidence of hippocampal atrophy, decreased parahippocampal thickness, and lower episodic memory scores, even before the onset of cognitive impairment. [11]. NfL levels have been shown to predict disease progression and prognosis across multiple conditions [12,13]. In the context of AD, NfL serves as a crucial biomarker alongside phosphorylated tau (p-Tau) and amyloid-beta (Aβ). Studies have demonstrated that increased NfL levels in both serum and CSF are associated with neuronal and axonal damage in AD patients, often correlating with cognitive decline and disease severity. The combination of NfL with p-Tau and Aβ provides a more comprehensive understanding of the pathological processes involved in AD, enhancing diagnostic accuracy [14]. Furthermore, the relationship between NfL and p-Tau is particularly noteworthy. While NfL reflects general neuroaxonal damage, p-Tau is more specific to tauopathies, including AD and frontotemporal dementia. The combination of elevated p-Tau and NfL levels has been shown to enhance the predictive accuracy for cognitive decline, indicating that these biomarkers may work synergistically to provide a clearer picture of neurodegenerative processes [15,16]. Particularly, tau protein phosphorylated at a threonine residue at position 181 (p-Tau 181) plasma levels are 3.5-fold increased in patients with AD as compared to controls, and this change is greater than the one of any other plasma biomarker [17]. Moreover, the relationship between these biomarkers and cognitive outcomes has been further elucidated in longitudinal studies. For example, research involving cognitively unimpaired individuals has shown that baseline levels of p-Tau and neurofilament light can predict subsequent cognitive decline over several years [18]. This predictive capability is crucial for early intervention strategies in at-risk populations. The present study investigated the relationship between hearing loss, cognitive impairment, and serum neurodegeneration biomarkers before and after hearing aid fitting. We studied the levels of NfL and p-Tau 181 in patients with ARHL, aiming to evaluate whether there is an alteration in these proteins' serum levels and if they could serve as markers of neuronal damage in patients with sensorineural hearing loss. Additionally, psychometric tests were used to evaluate various aspects of behavior function to create a patient’s cognitive strengths and weaknesses profile. These tests utilize a combination of standardized tests and questionnaires to assess different cognitive domains and their impact on daily life. All these data were then re-evaluated in the same patients after using the hearing aids for 6 months.

## 2. Materials and Methods

### 2.1. Participants

The study was conducted at the “AOU Gaetano Martino” University Hospital of Messina, including 50 participants, divided into two groups: 30 patients (17 males and 13 females) with age-related hearing loss and 20 age- and sex-matched healthy controls. The inclusion criteria for the patients group were as follows: age between 50 and 70 years, bilateral, symmetric sensorineural hearing loss with an air conduction pure-tone average at frequencies of 500, 1000, 2000, and 4000 Hz ranging from 35 to 64 dB HL (indicating a degree of hearing loss from moderate to moderately severe, according to the classification revision by the Global Burden of Disease Expert Group on Hearing Loss, 2011 [19]. For both groups: no family history of genetic hearing loss, neurodegenerative diseases, or dementia, and the absence of other pathological conditions, patients who had never been rehabilitated with hearing aids. Exclusion criteria included subjects with severe to profound bilateral sensorineural hearing loss, subjects undergoing pharmacological treatment with beta-blockers, neuroleptics, vasoactive agents (such as phosphodiesterase inhibitors), or sleep medications (such as benzodiazepines), subjects with other ear pathologies (Table 1).

### 2.2. Audiological Evaluation

The routine tests conducted at the center include pure-tone audiometry and speech audiometry, both performed in a soundproof booth capable of reducing ambient noise by approximately 40 dB SPL. For these assessments, TDH39 headphones and the B71 bone vibrator are used for pure-tone audiometry, and TDH39 headphones are used for speech audiometry. All testing is carried out with the Madsen Astera2 audiometer (Otosuite V. 8.84.0 software). Pure-tone audiometry is used to determine the minimum hearing threshold for both air and bone conduction, while speech audiometry evaluates the patient’s ability to understand speech sounds. The Air Conduction Pure-Tone Average (AC PTA) and the Bone Conduction Pure-Tone Average (BC PTA) are calculated, with the AC PTA representing the mean of the air conduction hearing thresholds at 500, 1000, 2000, and 4000 Hz, and the BC PTA representing the mean of the bone conduction hearing thresholds at the same frequencies. Speech audiometry is performed using phonetically balanced disyllabic words in Italian. The percentage of words correctly repeated by the patient from a list of 10 words is defined as the Word Recognition Score (WRS). The Matrix Sentence Test is an adaptive speech audiometry tool that replicates challenging listening environments by introducing speech messages embedded in background noise, simulating real-world situations [20]. This test determines the Speech Reception Threshold (SRT), expressed in dB SNR, which represents the signal-to-noise ratio at which the patient can correctly repeat 50% of the presented speech material. The test involves 20 randomly generated sentences, each consisting of five words. Noise is fixed at 65 dB SPL, while the speech level is adapted according to the patient’s responses. As one of the most effective tools for assessing hearing aid outcomes, the Matrix Sentence Test forms the basis for evaluating functional results and auditory outcomes (Table 2).

### 2.3. Hearing Aids

The Widex Moment RIC-312 models (220, 330, and 440) were used as hearing aids in this study, all belonging to the Receiver-In-Canal (RIC) category. Receiver power types M, P, and HP were selected based on the individual’s audiometric profile and the fitting software’s recommendations. Similarly, the coupling system (open dome, tulip dome, or closed dome) was chosen in accordance with the software’s suggestions to ensure the prescribed amplification targets were achieved. The NAL-NL2 prescriptive method was employed. Hearing aid selection was a collaborative process with the patient, considering factors such as hearing loss severity, cost, esthetics, ease of use, and maintenance. Audiogram data were uploaded into the fitting software (Widex Compass GPS V.4) to generate the target amplification curve—initial setup included in situ pure-tone audiometry (Sensogram) and anti-feedback calibration. The devices were configured with full noise reduction and listening optimization features to enhance the listening experience. Patients attended weekly follow-up sessions during the first four weeks to allow gradual amplification adjustments and facilitate acclimatization to the hearing aids [21].

### 2.4. Cognitive Evaluation

This evaluation delves into various aspects of mental function to create a detailed profile of a patient’s cognitive strengths and weaknesses. It utilizes a combination of standardized tests and questionnaires to assess different cognitive domains and their impact on daily life. The following tests have been used in this work.

#### 2.4.1. Overall Cognitive Screening (Mini-Mental State Examination—MMSE)

Mini-Mental State Examination (MMSE) is a widely used tool in clinical practice [22]. The MMSE assesses orientation in time and space, immediate and delayed recall of words, attention and calculation abilities, language fluency, and visuospatial skills through a series of simple tasks. Scores range from 0 (indicating severe cognitive impairment) to 30 (reflecting normal cognitive function). A score below 23 on the MMSE may suggest potential cognitive decline and warrant further investigation [23].

#### 2.4.2. Detailed Memory Assessment

Memory function is often a primary area of interest, and two commonly used tests provide valuable insights.

- Rey Auditory Verbal Learning Task (RAVLT): This test assesses a patient’s ability to encode, store, and retrieve verbal information. Participants are presented with a list of words to remember and asked to recall them immediately and again after a short delay (typically 15 min). The RAVLT not only evaluates immediate and delayed recall (episodic memory) but also may involve some planning skills during the initial learning phase [24].
- Rey-Osterreith Complex Figure Test: This test goes beyond verbal memory and assesses a patient’s long-term visual memory and other cognitive functions like visuospatial abilities and planning]. Participants are asked to copy a complex line drawing figure and then to draw it again from memory after a delay (usually 15 min). The performance of this test reflects not only the ability to retain visual information but also the ability to plan and execute the drawing based on memory [24].

#### 2.4.3. Executive Function Evaluation (Mental Flexibility and Processing)

Executive functions are a set of higher-order cognitive skills crucial for planning, organizing, focusing attention, and problem-solving. Several tests can assess different aspects of executive function during this evaluation.

- Digit Span Forward and Backward Test: This test challenges a core aspect of working memory, the ability to hold and manipulate information in the mind for a short period. Performance on the Digit Span Forward and Backward Test can provide insights into a patient’s attention to detail, auditory processing speed, and ability to mentally manipulate information [25].
- Corsi Block-Tapping Test: Similar to the Digit Span Test, but instead of numbers, this test uses a sequence of taps on spatially arranged blocks that the patient must replicate in the same order (forward) or reverse order (backward) [26]. This task assesses visuospatial working memory, which is crucial for activities like navigating unfamiliar environments and following complex instructions.
- Trail-Making Test: This test evaluates a combination of cognitive skills, including visual attention, processing speed, and mental flexibility [27]. Participants are presented with a sheet containing circles numbered consecutively or alternating numbers and letters. The task is to connect the circles in the correct sequence as quickly and accurately as possible. Performance on different parts of the Trail-Making Test reflects a patient’s ability to sustain attention, switch focus rapidly, and adapt to changing task demands.
- WEIGL Test: The WEIGL test evaluates the abstraction process and the flexibility of thinking to make different categorizations. The test is made up of 12 pieces of wood that differ in shape (4 circles, 4 squares, and 4 triangles), color (3 red, 3 yellow, 3 blue, and 3 green), suits (4 with hearts, 4 with diamonds and 4 with flowers), size and thickness [28]. The test is carried out in time according to an active mode and a passive mode: in the first case, the subject is asked to categorize the stimuli on the basis of perceptive characteristics, then to divide the pieces of wood by identifying the 5 expected characteristics one at a time; in the second case, however, it is the examiner who divides the pieces, and the subject must indicate the criterion for the division. The scoring application allows you to transform the raw score obtained by the subject into a correct score and the corresponding equivalent score.
- Phonological Word Fluency Test: This test assesses a combination of verbal fluency, semantic knowledge, and mental flexibility. Patients are given a limited time to generate as many words as possible that begin with a specific letter. This task requires patients to search their memory for words, retrieve relevant options, and maintain mental flexibility by avoiding repetitions while generating new words within the given time limit. Performance on the Phonological Word Fluency Test can provide clues about a patient’s language skills, access to semantic memory, and ability to think creatively [29].

#### 2.4.4. Quality of Life Assessment

Patients’ quality of life was assessed using the SF-36 questionnaire [30]. This 36-question survey covers eight key areas of health: physical functioning, limitations due to physical health, pain, general health, energy, social activities, emotional limitations, and mental health.

### 2.5. Serum NfL and p-Tau 181 Quantification

Centrifuged plasma aliquoted in polypropylene tubes and stored at −80 °C was used to measure NfL and p-tau 181 using SIMOA technology (Quanterix, Billerica, MA, USA). SIMOA technology identifies and quantifies immunocomplexes bound to dye-encoding magnetic beads (using different capture and detector antibodies) sealed in arrays of femtoliter-volume microwells. Rapid-thawed plasma was centrifuged at 10,000× *g* for 10 min, 25 μL (diluted 4-fold in buffer) was added to kit beads (100 μL) by pipette in each well, the plate was incubated for 15 min at 30 °C, magnetic-washed 3× for 5 min total, subjected to addition of SBG reagent (100 μL), followed by another incubation for 10 min at 30 °C, washed again × 5 for 7 min total, and reading on the SIMOA SR-X machine. All measurements were performed in duplicate to assure inter-assay precision by calculating the coefficient of variation (CV). Results with CVs below 20% were accepted for statistical analysis.

### 2.6. Procedure

In the initial phase, the subjects underwent an otomicroscopic examination to exclude the presence of earwax, acute inflammatory processes, or any malformations and abnormalities of the outer and middle ear. Following this, three audiometric tests (pure-tone and speech audiometry) were conducted, spaced two months apart, to ensure the stability of the hearing threshold and exclude cases of fluctuating hearing loss. The initial audiological assessment included pure-tone audiometry, speech audiometry, and the Matrix Sentence Test conducted in a free field prior to the fitting of the hearing aid. Once age-related hearing loss was confirmed, the patients underwent venous blood sampling to measure plasma levels of NfL and p-Tau. Cognitive evaluation tests were also administered during the same session. Following this, the hearing aid was selected and fitted to the patient. The hearing aid was programmed to utilize all available noise reduction and listening optimization features to enhance the listening experience. Weekly follow-up appointments were conducted during the first four weeks to gradually adjust the amplification gain, ensuring the patient adapts comfortably to the provided amplification. Starting from the fifth week, real-ear measurements (REM) were conducted for patients who had reached the estimated target. The REM procedure was carried out using the AURICAL FreeFit system (Otosuite software V. 8.84.0) in accordance with EN 61669:2016 standards [31], with the equipment calibrated within the previous 12 months. These measurements are used to determine whether the hearing aid provides an output in dB SPL appropriate for the patient’s hearing loss by comparing the target amplification gain curve to the actual gain measured inside the patient’s ear canal while the hearing aid is powered on and functioning. The same procedure described by Portelli et al. was carried out [32]. Minimal adjustments between the real and target curves were made to ensure complete alignment between them. In the sixth month, the Matrix Sentence Test was conducted in a free field with the hearing aids powered on to assess the auditory outcomes of these patients. Additionally, serum levels of NfL and p-Tau were re-measured, and MMSE was repeated.

### 2.7. Statistical Analysis

First, descriptive statistics for participants’ characteristics were tabulated. Then, the Mann–Whitney test was used to compare the differences between groups. Furthermore, Spearman’s rank correlation coefficient was calculated to look for a correlation between MMSE and SRT test scores and serum biomarker values. Statistical analysis was conducted using GraphPad PRISM software 9.5.1. *p* values less than 0.05 were considered to indicate statistical significance.

## 3. Results

### 3.1. Cognitive Performance

**Figure 1:**
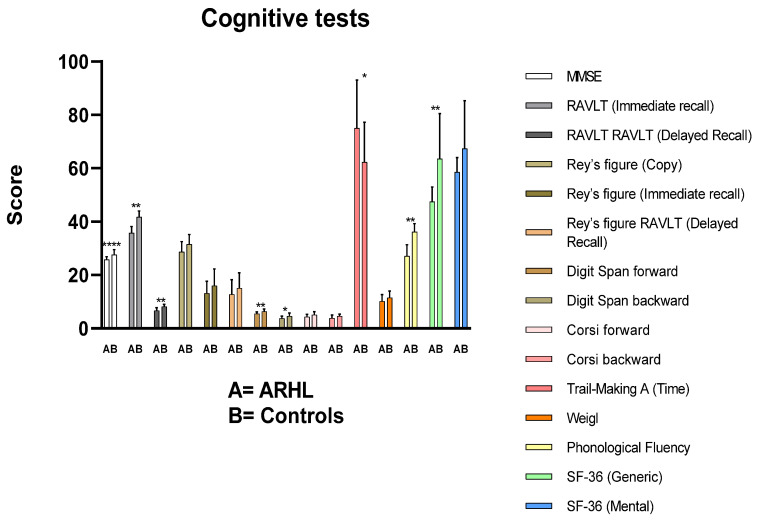
Cognitive tests score before hearing aids. Mann–Whitney test. Data are expressed as means ± standard deviations. * = *p* < 0.05; ** = *p* < 0.005; **** = *p* < 0.0001. ARHL, Age-related hearing loss group; MMSE, Mini-Mental State Examination; RAVLT, Rey Auditory Verbal Learning Task.

Results are summarized in Figure 1. The general cognitive status of ARHL has been evaluated by the Mini-Mental State Examination—MMSE. As expected, according to the literature [22], it appears that the cognitive performance of ARHL patients was influenced by their status as evidenced by the lower values obtained with respect to Controls (25.8 and 27.6, respectively, *p* < 0.0001); however, their performance was higher than common cutoff value considered for this test (24.90). Indeed, MMSE could not be able to evidence cognitive decline related to specific abilities such as processing speed and access to working memory. Significant differences (*p* < 0.005) in short and long-term memory performance characterize ARHL subjects when compared to Controls. Once again, their performance is, however, within the normality status if we refer to cutoff values (<28.53 for RAVLT immediate recall and <4.69 for RAVLT delayed recall) taken from the literature [24]. Hand executive functions and visuospatial working memory (evaluated employing Rey’s figure test and Corsi Block-Tapping Test) are more related to the prefrontal cortex and frontal lobes function seems to be not influenced by hearing loss status (*p* > 0.05) Digit Span Forward (5.5 vs. 6.4, *p* < 0.005), and Backward (3.8 vs. 4.6, *p* < 0.05) tests allowed to evidence a noteworthy influence of hearing loss status on cognitive abilities and, in particular, on working memory. Speech-related evaluation tests (phonological fluency, cutoff value 17.35) highlight difficulties characterizing ARHL patients in such tasks (*p* < 0.005). Also, the Trail Making Test A, being related to working memory and attention as well as information processing, evidences a slight cognitive decline of ARHL subjects (75 vs. 62.3, *p* < 0.05).

### 3.2. Biomarkers

**Figure 2:**
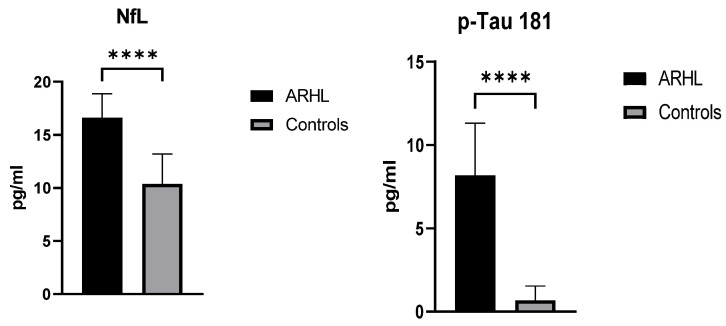
Biomarkers level before hearing aids. Mann–Whitney test. Data are expressed as means ± standard deviations. **** = *p* < 0.0001. ARHL, Age-related hearing loss group.

Serum NfL and p-Tau 181 levels were determined by SIMOA in ARHL and Control groups. Both biomarkers’ levels were statistically significantly higher in the ARHL group compared to the control group (*p* < 0.0001) (Figure 2).

**Figure 3:**
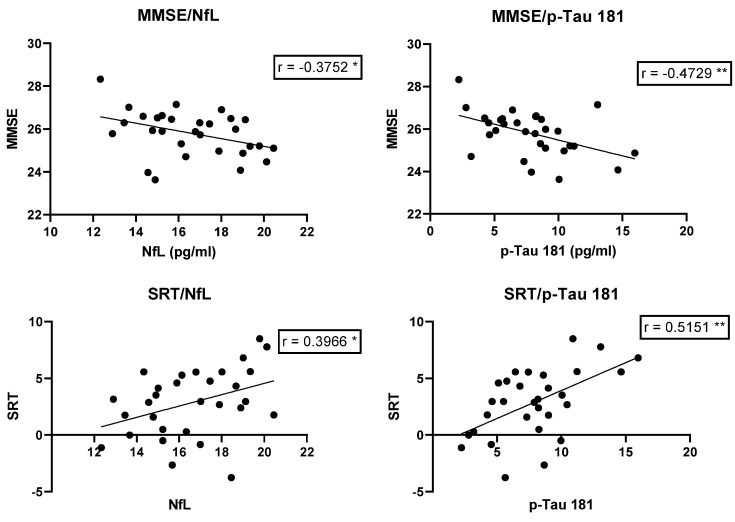
MMSE and SRT score/biomarkers correlations in ARHL group before hearing aids. Spearman’s rank correlation coefficient. * = *p* < 0.05; ** = *p* < 0.005; ARHL, Age-related hearing loss group; MMSE, Mini-Mental State Examination; SRT, Speech Reception Threshold.

The highly significant increase in the two biomarkers in the serum of the ARHL group highlights the possible neurodegenerative cause of MCI contextual to hearing loss. To strengthen this theory, we also evaluated the correlation between the two biomarkers levels and the scores of MMSE and SRT, tests that evaluate word comprehension and general cognitive status: there is a highly significant correlation between biomarkers and test scores; therefore, biomarkers' high values correspond to worse test values (Table 3, Figure 3).

**Figure 4:**
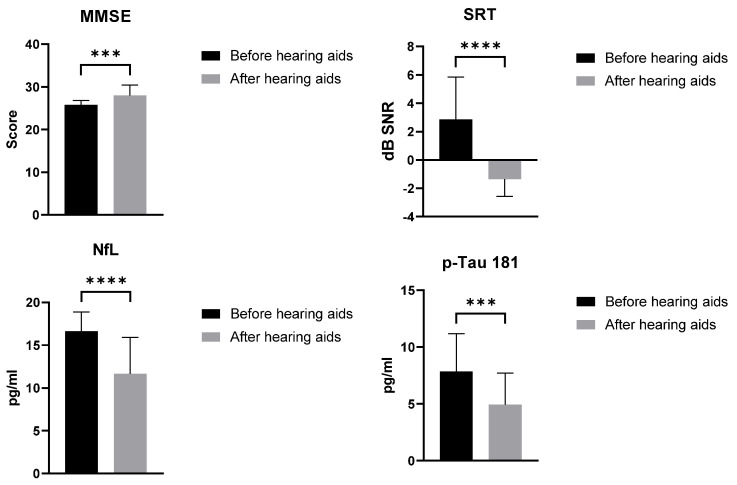
ARHL group valuation after hearing aids use. Mann–Whitney test. Data are expressed as means ± standard deviations. *** = *p* < 0.0005; **** = *p* < 0.0001. ARHL, Age-related hearing loss group; MMSE, Mini-Mental State Examination; SRT, Speech Reception Threshold.

After six months of hearing aid use, we re-evaluated the MMSE and SRT scores and serum biomarkers levels in the ARHL group: we recorded significant improvements both in cognitive and word recognition tests and in biomarker values (Figure 4). We also re-evaluated the biomarker levels in the control group after six months and found no significant changes.

## 4. Discussion

ARHL is a disorder characterized by a progressive deterioration in hearing ability over time, resulting from the cumulative effects of aging on the auditory system. The hearing loss is sensorineural, bilateral, and symmetrical and can vary in degree. This involves the gradual degeneration of outer and inner hair cells, alterations in the stria vascularis, and degeneration of nerve fibers; these refer to sensory presbycusis, strial presbycusis, and neural presbycusis, respectively. These changes are exacerbated by alterations in the central auditory pathways, contributing to the progressive development of ARHL. From an etiopathogenetic perspective, ARHL is a multifactorial disorder caused by both modifiable and non-modifiable factors such as age, sex, race, noise exposure, ototoxic medications, lifestyle, comorbidities, and genetics, making the condition clinically variable [3,33].

Numerous studies have highlighted how hearing loss can lead to social isolation, loss of self-esteem, reduced quality of life, and an increased risk of psychiatric disorders [25]. While the use of hearing aids can mitigate these issues, the adoption rate of hearing aids remains low [34]. The precise mechanism by which hearing loss leads to dementia is not yet fully understood, but it has been observed that there is a reduction in the volume of the auditory cortex, along with atrophy of the superior, middle, and inferior temporal gyri and the parahippocampal gyrus, all areas commonly affected in AD. Additionally, histopathological changes typical of AD, such as degeneration, plaques, and tangles, are found throughout the auditory pathway [35,36,37].

Excessive cognitive load dedicated to auditory perceptual processing in daily life induces significant structural brain changes and neurodegeneration at the expense of other cognitive processes, creating a vicious cycle where available cognitive resources for auditory perception are diminished. The cognitive load in deaf individuals diverts cognitive resources from other processes, such as working memory, which could hypothetically lead to cognitive decline. Individuals with hearing loss experience a 30–40% faster rate of cognitive decline [35,38].

However, dementia may result from social isolation or cognitive load [39]. In relation to this, four hypotheses regarding the relationship between cognitive capacity and cognitive processing load (effort) have been proposed [38]. The “effort hypothesis” assumes that a person with high cognitive ability employs more resources to perform a task, achieving better performance with a greater cognitive processing load, regardless of difficulty. The “cognitive efficiency hypothesis” suggests that individuals with high cognitive ability use their capacity more efficiently, thereby reducing the processing load regardless of listening difficulty. The “resource hypothesis” states that individual differences in capacity affect speech perception, especially in challenging listening environments; in such situations, greater capacity allocation improves performance and processing load. Thus, higher cognitive abilities are associated with a higher load, particularly in competitive conditions [40,41]. The final hypothesis, the “ease-of-language understanding” (ELU model), theorizes that cognitive abilities and working memory play a key role in challenging listening conditions. In difficult listening situations, the cognitive processing load is lower for individuals with better working memory. Hence, higher cognitive abilities are correlated with a lower cognitive load in competitive conditions [42].

Research indicates that ARHL is often accompanied by neurodegenerative changes in the auditory pathways and associated brain regions. For instance, Ohgami et al. demonstrated that manganese exposure exacerbates age-related hearing loss in mice, leading to neurodegeneration of spiral ganglion neurons and impairment of critical signaling pathways such as c-Ret and NF-κB [43]. This finding underscores the potential for environmental factors to influence both hearing and neurodegenerative processes. Moreover, studies have shown that reduced auditory input due to hearing loss can lead to hippocampal degeneration and impaired cognitive functions such as memory, and they found that mice with age-related hearing loss exhibited significant hippocampal degeneration, correlating with deficits in spatial memory and that these alterations may be the first evidence of AD [44,45]. This suggests that the auditory system’s health is intricately linked to cognitive functions, particularly in aging populations. The inflammatory processes associated with aging, termed “inflammaging,” have also been implicated in both ARHL and neurodegenerative diseases, highlighting the role of inflammation in cochlear damage, suggesting that dietary factors influencing inflammation may be linked to the onset of hearing loss [46]. Similarly, Kociszewska et al. discussed how immunosenescence contributes to ARHL, indicating that age-related changes in the immune system may exacerbate neurodegenerative conditions [47]. Furthermore, the relationship between hearing loss and cognitive decline has been supported by epidemiological studies that showed that hearing impairment is associated with an increased risk of cognitive impairment and dementia, particularly highlighting the role of neurodegeneration in this association [48]. Common neurodegenerative mechanisms shared by hearing loss and cognitive decline could be a significant factor in the observed correlations [49]. The potential for ARHL to serve as a modifiable risk factor for dementia has been evaluated: addressing hearing loss could reduce the risk of cognitive decline [50]. This is particularly relevant given that ARHL is one of the most common sensory disorders in older adults and affects their overall health and well-being [51].

Since the pathophysiology of ARHL involves various mechanisms, including oxidative stress, mitochondrial dysfunction, and neuroinflammation [52], acoustic neuronal damage and cerebral neuronal damage due to cognitive decline could be revealed by serum neurodegeneration biomarkers as NfL and p-Tau 181, to understanding the neurodegenerative processes associated with ARHL. Elevated levels of these proteins have been linked to neurodegenerative diseases such as AD, suggesting a possible common biomarker between ARHL and cognitive decline [53]. Furthermore, ARHL was associated with higher levels of CSF total tau and p-Tau 181, and neuropathological and in vivo studies indicated that tau pathology could induce cognitive impairment across the AD spectrum via synaptic dysfunction and neuronal loss [54,55].

The mechanisms linking ARHL to elevated NfL and p-Tau 181 levels may involve several pathways. Oxidative stress has been implicated in both ARHL and neurodegenerative diseases, leading to mitochondrial dysfunction and neuronal damage [56]. Additionally, neuroinflammatory processes, characterized by increased levels of pro-inflammatory cytokines, have been observed in the cochlea of individuals with ARHL, potentially contributing to neuronal degeneration [57].

Furthermore, the cognitive load associated with hearing loss may exacerbate neurodegenerative processes. Individuals with ARHL often experience increased cognitive strain due to difficulties in auditory processing, which may lead to greater neuronal stress and subsequent elevation of neurodegenerative biomarkers [58]. Research indicates that it is, above all, the frontal lobe that is stressed. This increased cognitive effort can adversely affect memory and executive function, which are primarily managed by the frontal lobe [59]. This relationship underscores the need for comprehensive assessments of auditory and cognitive health in older adults.

Our preliminary data in a small cohort of patients suggest that the presence of signs of mild cognitive impairment (MCI) in patients with ARHL is related to high serum levels of NfL and p-Tau 181. In particular, there is a significant correlation between high levels of these biomarkers and cognitive and word recognition test scores of our patients. Further confirmation of the link between ARHL, MCI, and neurodegeneration was highlighted by the significant improvement in both the auditory/cognitive status and the NfL and p-Tau values 181. These findings confirm the association between ARHL and neurodegeneration, promoting these biomarkers as early indicators of cognitive decline, allowing for timely interventions aimed at mitigating the progression of both hearing loss and cognitive impairment [60].

These results highlight the involvement of p-Tau 181 and NfL in ARHL patients, leading the way to future research that should focus on longitudinal studies to establish causal relationships and explore the potential for therapeutic strategies targeting neuroinflammation and oxidative stress to preserve auditory function in aging populations. The exploration of neurofilament and p-Tau 181 serum levels in patients with age-related hearing loss presents a promising avenue for understanding the underlying neurodegenerative processes associated with this condition.

## 5. Conclusions

In summary, elevated levels of NfL and p-Tau 181 in serum are associated with ARHL and MCI, suggesting a potential link between neurodegeneration and auditory and cognitive decline. Furthermore, the correction of the hearing deficit not only improves auditory/cognitive performance but evidently has a direct positive effect on the neurodegenerative damage associated with ARHL, demonstrated by the drop in biomarker levels. Our study has a few limitations. First, although the literature underlines the evidence for a causal link between ARHL and MCI, and we excluded individuals with mental health or neurological diseases, we cannot entirely rule out the influence of other potential confounders, such as social isolation and depression that potentially can exacerbate cognitive decline influencing neurodegenerative biomarker profiles. Second, individuals with moderate to severe hearing loss may struggle to hear and process the verbal components of cognitive tests, such as MMSE, leading to an underestimation of their cognitive capabilities: future research should focus on using alternative non-verbal assessment methods. Third, the significance of improvements in MMSE scores compared to clinical improvements remains a topic of debate. While an increase in MMSE scores can indicate cognitive enhancement, the clinical relevance of such changes, particularly those less than 3 points, is often questioned. In future research, MMSE should be used in conjunction with other clinical assessments to provide a more comprehensive understanding of cognitive function and its implications for patient care. Fourth, the small size of the population requires a future increase in examined subjects to validate our results. In the next part of our study, it will be interesting to associate the levels of neurodegeneration biomarkers with the MRI evidence of subjects with AHRL to stage organic and cognitive damage more accurately. A better understanding and staging of the mechanisms underlying cognitive disorders in ARHL would allow a better therapeutic approach, given that the latest generation hearing aids allow targeted and effective interventions [61,62].

## Figures and Tables

**Table 1:** Demographic characteristics of study participants.

	ARHL	Controls
Number	30	20
Age, years	63.97 ± 8.14	60.40 ± 5.31
Sex, male, *n* (%)	17 (56)	10 (50)

Data are expressed as means ± standard deviations. ARHL, age-related hearing loss.

**Table 2:** Audiological tests before hearing aids fitting.

	ARHL	Controls	*p* Value
Right AC PTA (dB HL)	50.75 ± 7.15	11.63 ± 3.22	****
Left AC PTA (dB HL)	49.42 ± 7.83	13.25 ± 4.00	****
Right BC PTA (dB HL)	45.54 ± 6.80	6.63 ± 3.22	****
Left BC PTA (dB HL)	44.63 ± 8.26	8.25 ± 4.00	****
Right WRS (%)	93.67 ± 8.50	100	
dB WRS (dB HL)	56.67 ± 19.36	36.50 ± 4.89	**
Left WRS (%)	92.33 ± 9.71	100	
dB WRS (dB HL)	57.00 ± 19.50	37.00 ± 4.70	****
SRT (dB SNR)	2.79 ± 3.03	−3.7 ± 2.22	****

Mann–Whitney test. Data are expressed as means ± standard deviations. ** = *p* < 0.005; **** = *p* < 0.0001. AC PTA, Air Conduction Pure-Tone Average; BC PTA, Bone Conduction Pure-Tone Average; WRS, Word Recognition Score; SRT, Speech Reception Threshold; SNR, Signal to Noise Ratio.

**Table 3:** MMSE and SRT score/biomarkers correlations in ARHL group before hearing aids.

	r	*p*
MMSE/p-Tau 181	−0.4279	**
MMSE/Nfl	−0.3752	*
SRT (dB SNR)/p-Tau 181	0.5151	**
SRT (dB SNR)/NfL	0.3966	*

Spearman’s rank correlation coefficient. * = *p* < 0.05; ** = *p* < 0.005; ARHL, Age-related hearing loss group; MMSE, Mini-Mental State Examination; SRT, Speech Reception Threshold; SNR, Signal to Noise Ratio.

## Data Availability

The raw data supporting the conclusions of this article will be made available by the authors upon request.
